# Maternal Protein Restriction Affects Postnatal Growth and the Expression of Key Proteins Involved in Lifespan Regulation in Mice

**DOI:** 10.1371/journal.pone.0004950

**Published:** 2009-03-24

**Authors:** Jian-Hua Chen, Malgorzata S. Martin-Gronert, Jane Tarry-Adkins, Susan E. Ozanne

**Affiliations:** University of Cambridge Metabolic Research Laboratories Institute of Metabolic Science, Addenbrooke's Hospital, Cambridge, United Kingdom; Sapienza University of Rome, Italy

## Abstract

We previously reported that maternal protein restriction in rodents influenced the rate of growth in early life and ultimately affected longevity. Low birth weight caused by maternal protein restriction followed by catch-up growth (recuperated animals) was associated with shortened lifespan whereas protein restriction and slow growth during lactation (postnatal low protein: PLP animals) increased lifespan. We aim to explore the mechanistic basis by which these differences arise. Here we investigated effects of maternal diet on organ growth, metabolic parameters and the expression of insulin/IGF1 signalling proteins and Sirt1 in muscle of male mice at weaning. PLP mice which experienced protein restriction during lactation had lower fasting glucose (*P* = 0.038) and insulin levels (*P* = 0.046) suggesting improved insulin sensitivity. PLP mice had higher relative weights (adjusted by body weight) of brain (*P* = 0.0002) and thymus (*P* = 0.031) compared to controls suggesting that enhanced functional capacity of these two tissues is beneficial to longevity. They also had increased expression of insulin receptor substrate 1 (*P* = 0.021) and protein kinase C zeta (*P* = 0.046). Recuperated animals expressed decreased levels of many insulin signalling proteins including PI3 kinase subunits p85α (*P* = 0.018), p110β (*P* = 0.048) and protein kinase C zeta (*P* = 0.006) which may predispose these animals to insulin resistance. Sirt1 protein expression was reduced in recuperated offspring. These observations suggest that maternal protein restriction can affect major metabolic pathways implicated in regulation of lifespan at a young age which may explain the impact of maternal diet on longevity.

## Introduction

It is well established that nutrition can affect organismal lifespan. Caloric restriction (CR) is one of the most extensively studied feeding regimes shown to influence lifespan. Since the first report by McCay *et al* that restricting food intake of rats markedly extended their mean and maximal lifespan [1] CR has been proven to be a robust feeding regime for lifespan extension in a wide range of model organisms including yeast, invertebrate animals and many mammalian species [2].

We have reported previously that changes in nutrition during fetal or early postnatal life alone are sufficient to have marked effects on lifespan in rats and mice [3], [4], [5]. Offspring born to normally fed dams but suckled by protein restricted dams (postnatal low protein: PLP animals) grew slowly during lactation and exhibited significantly longer lifespan when fed *ad libitum* on standard chow. Conversely offspring born to protein restricted dams but suckled by normally fed dams (recuperated animals) were smaller at birth, showed rapid catch-up growth and had a reduced longevity when fed *ad libitum* on standard chow. These findings demonstrate that nutrition during critical periods of development has a major impact on longevity. These findings are consistent with the developmental origins of health and disease hypothesis which suggests that the pre- and postnatal environment may program health/disease outcomes in adult life [6], [7]. This hypothesis is based on human and animal studies linking early growth and nutrition to long term risk of age-related diseases such as type 2 diabetes [8], [9], [10]. There is also limited data suggesting a link between early life events and the aging process in humans [11]. However, the molecular mechanism underlying such an association is not understood.

Studies using model organisms ranging from worms and flies to mammals have revealed that the insulin/insulin-like growth factor-1 (IGF1) signalling pathway is a highly conserved mechanism that influences lifespan [12], [13], [14], [15], [16]. It was first demonstrated in *C. elegans* that mutation in *age-1*, a homologue of the mammalian phosphatidylinositol-3 kinase (PI3 kinase) [17] extended lifespan [18], [19]. Subsequently it was found that a mutation in *daf-2*, a homologue of the mammalian insulin and IGF1 receptors [20], also dramatically prolonged the lifespan of *C. elegans* [21]. Similarly, reduced insulin signalling was demonstrated to extend lifespan in *Drosophila* [14]. The involvement of insulin/IGF1 signalling in lifespan regulation in mammalian species was first suggested in Ames and Snell dwarf mice in which insulin/IGF1 function is reduced due to a deficiency in growth hormone [22]. Recent observations in knockout mice further provided evidence for a direct role of reduced insulin/IGF1 signalling in regulation of mammalian lifespan. Female mice heterozygous knockout for IGF1 receptor exhibited a long-lived phenotype [23], [24]. Fat-specific insulin receptor knockout mice had a better maintenance of mitochondrial activity in adipose tissue and lived longer than their littermates [25], [26]. Insulin receptor substrate 1 (IRS1) null female mice showed increased lifespan which was accompanied by a reduction in markers of aging [27].

The mechanisms by which caloric restriction leads to increased longevity are not fully understood. However it has been suggested that the lifetime maintenance of low levels of glucose and insulin may play a major role [2]. Up-regulation of Sirt1, an NAD-dependent deacetylase is also thought to play a pivotal role in mediating lifespan extension by CR [28]. Transgenic mice overexpressing Sirt1 have a phenotype that resembles that of CR [29] whereas Sirt1-null mice fail to show the normal metabolic response to CR and their lifespan can no longer be extended by CR [30].

We aim to understand the mechanistic basis through which maternal diet influences lifespan in mice. In this study we subjected mice to exactly the same maternal protein restriction regime that is known to influence lifespan and investigated the effects of maternal protein restriction on metabolic parameters, organ growth and expression of insulin signalling proteins and Sirt1 in muscle from mouse offspring at weaning. We focused on this young age is order to identify potential early mechanisms underlying the association between maternal diet and offspring lifespan. Here we report the changes at the tissue and molecular levels which may explain the impact of maternal diet on longevity.

## Results

### Body weights

**Figure 1:**
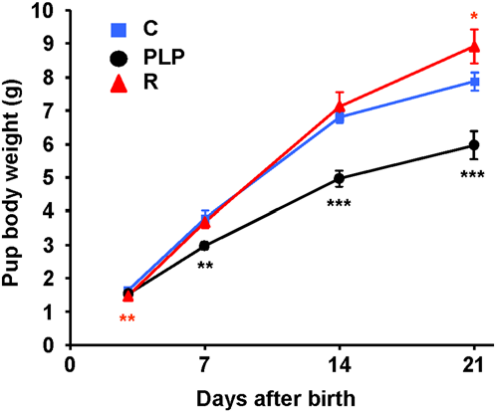
Growth curves of pups of control, postnatal low protein and recuperated mice during lactation. Body weights of pups were recorded at days 3, 7, 14 and 21 of age. To maximize the effects of maternal diet, recuperated pups (R) were culled to 4 and control pups (C) were culled to 8 (if litter size was greater than 8) whereas postnatal low protein pups (PLP) were unculled. Means±SEM are shown (* *P*<0.05, ** *P*<0.01, *** *P*<0.001 compared to control; n = C: 13, PLP: 11, R: 16).

Pups of mothers fed the low protein diet during pregnancy were significantly smaller than controls (1.46±0.04 g vs 1.65±0.07 g on day 3, *P* = 0.006; Figure 1). Cross-fostering to mothers fed the control diet resulted in rapid catch-up growth such that by day 7 recuperated offspring had a similar body weight to controls (3.68±0.18 g vs 3.78±0.22 g; Figure 1), by day 14 they had overtaken the weight of controls and at day 21 they were significantly heavier than controls (8.92±0.52 g vs. 7.89±0.26 g, *P* = 0.047; Figure 1). In contrast, offspring of normally fed mothers when suckled by low protein fed dams grew slowly during lactation; by day 7 they were significantly smaller than controls (2.98±0.11 g vs. 3.78±0.22, *P* = 0.002; Figure 1) and this difference was further increased by day 14 and day 21 (4.96±0.23 g vs. 6.79±0.15 g, *P* = 0.00013 and 5.96±0.42 g vs. 7.89±0.26 g, *P* = 0.0002; Figure 1).

### Organ weights

**Figure 2:**
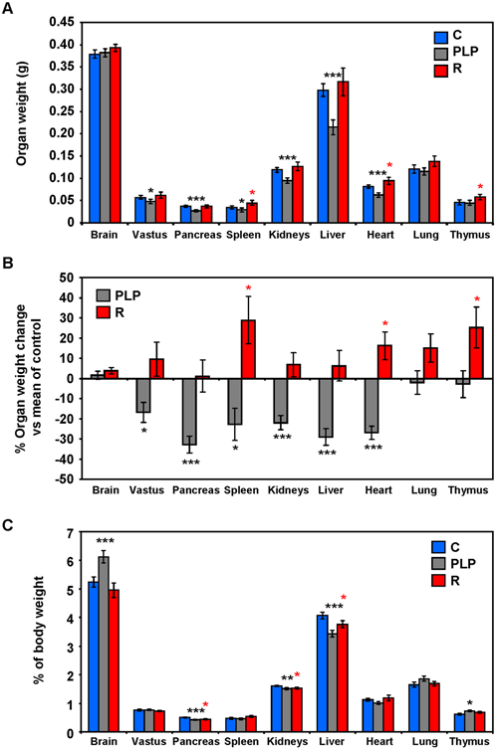
Effects of maternal protein restriction on organ weights. Organ weights were measured 21 days after birth. (A) Organ weights were expressed as mean±SEM (* *P*<0.05, ** *P*<0.01, *** *P*<0.001 compared to control; n = control (C): 10, postnatal low protein (PLP): 8, recuperated (R): 10). (B) Percentage organ weight change of PLP and R mice compared to the mean of control organ weights (mean±SEM; * *P*<0.05, ** *P*<0.01, *** *P*<0.001; n = C: 10, PLP: 8, R: 10). (C) Relative organ weights were expressed as percentage of body weights (mean±SEM; * *P*<0.05, ** *P*<0.01, *** *P*<0.001 compared to control; n = C: 10, PLP: 8, R: 10).

In general, except for the brain, lung and thymus, organ weights of PLP mice were smaller than that of control mice with vastus lateralis, pancreas, spleen, kidneys, liver and heart being significantly lighter (Figures 2A and 2B). In contrast, organs of recuperated mice were larger than that of control mice with spleen, heart and thymus being significantly heavier (Figures 2A and 2B). When expressed relative to body weight, PLP pancreas, kidneys and liver still remained significantly smaller than controls (Figure 2C). In contrast, the relative weights of brain and thymus in PLP were significantly higher than controls (Figure 2C). In recuperated animals the relative weights of pancreas, kidneys and liver were lower than controls, however the relative weights of spleen, heart, lung and thymus showed no difference as compared to controls (Figure 2C).

### Fasting glucose and insulin concentrations

**Figure 3:**
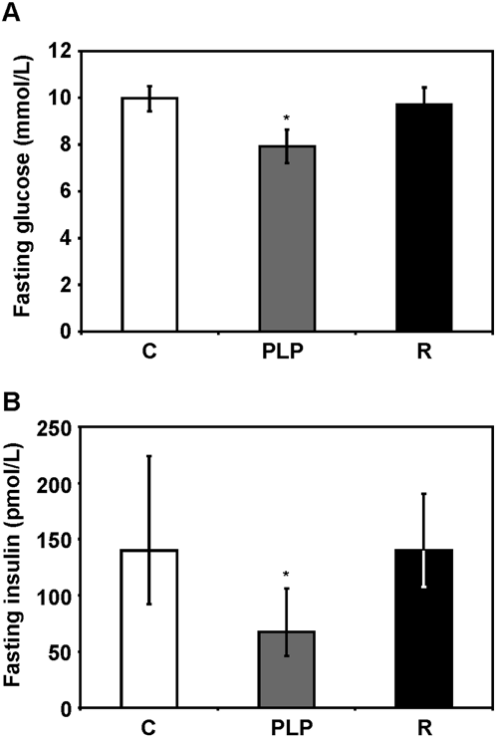
Fasting glucose and insulin concentrations. (A) Fasting blood glucose was measured using a blood glucose analyzer (Hemocue, Angelholm, Sweden). Values are expressed as mean±SEM, * *P*<0.05 compared to control (n = control (C): 10, postnatal low protein (PLP): 8, recuperated (R): 10). (B) Fasting blood insulin concentrations were measured using a mouse insulin ELISA kit (Mercodia Ultra-sensitive Mouse Insulin ELISA, Mercodia, Uppsala, Sweden). Values are expressed as geometric mean (95% confidence intervals) and was log transformed prior to analysis. * *P*<0.05 compared to control (n = C: 10, PLP: 8, R: 10).

Fasting blood glucose concentrations in control and recuperated mice were similar; however, fasting glucose was significantly lower in PLP animals compared to controls (*P* = 0.038, Figure 3A). Similarly, fasting blood insulin concentrations in control and recuperated mice showed no difference whereas the insulin levels in PLP animals were significantly lower than in the control group (*P* = 0.046, Figure 3B).

### Protein expression of insulin signalling molecules

**Figure 4:**
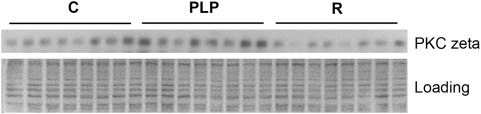
Representative Western blot for PKCζ in muscle. Protein levels of insulin signalling molecules in muscle of control (C), postnatal low protein (PLP) and recuperated (R) were analysed by Western blotting using appropriate antibodies. Shown is a representative blot for PKCζ. 25 µg of protein were used and equal loading was confirmed by Coomassie blue staining. Densitometry analysis data are summarized in Table 1.

We systematically analyzed by Western blotting the expression levels of proteins in the insulin signalling pathway in skeletal muscle, one of the major target tissues of insulin action and the major site of postprandial glucose disposal. Figure 4 shows a representative blot for PKCζ with densitometry data (arbitrary units) of blots for all the signalling proteins analyzed being summarized in Table 1. Maternal diet had profound effects on the expression levels of these proteins in PLP and recuperated mice. Specifically, protein restriction during lactation resulted in significant up-regulation of IRS1 (17% increase compared to controls, *P* = 0.021) and PKCζ (22% increase compared to controls, *P* = 0.046) (Table 1). In addition, postnatal protein restriction caused significant reduction in phosphorylation levels of IRS1 on Tyr^612^ (37% decrease compared to controls, *P* = 0.036) and Ser^307^ (79% decrease compared to control, *P* = 0.00007), and phosphorylation of Akt on Ser^473^ (69% decrease compared to controls, *P* = 0.00013) (Table 1). In contrast, recuperated mice showed a significant decrease in the protein expression levels of IGF1 receptor (30% decrease compared to controls, *P* = 0.007), p85α (30% decrease, *P* = 0.018), p110β (19% decrease, *P* = 0.048) and PKCζ (35% decrease, *P* = 0.006) (Table 1). Recuperated mice also showed significant decreased phosphorylation of IRS1 on Try^612^ (32% decrease compared to controls, *P* = 0.021) and Ser^307^ (34% decrease, *P* = 0.004), and phosphorylation of Akt on Ser^473^ (73% decrease, *P* = 0.0009) (Table 1).

**Table 1:** Expression levels of insulin signalling proteins in muscle.

Protein	Muscle		
	C (n = 8)	PLP (n = 8)	R (n = 8)
IGF1R	100±4	104±10	70±8 **
IRβ	100±8	109±5	89±8
IRS1	100±5	117±2 *	87±6
IRS1 (Tyr612)	100±10	63±14 *	68±11 *
IRS1 (Ser307)	100±10	21±5 ***	66±5 **
p85	100±8	110±16	70±8 *
p110β	100±6	81±11	81±9 *
PKCζ	100±9	122±11 *	65±9 **
Akt1	100±5	108±6	95±6
Akt2	100±7	91±11	84±6
Akt (Ser473)	100±21	31±9 ***	27±7 ***

Expression levels of insulin signalling proteins were analysed by Western blotting (see Figure 4) followed by densitometry determination and are expressed as mean±SEM. The number of mice used for each group is shown in parenthesis.

* *P*<0.05.

** *P*<0.01.

*** *P*<0.001 compared to control.

### Sirt1 expression

**Figure 5:**
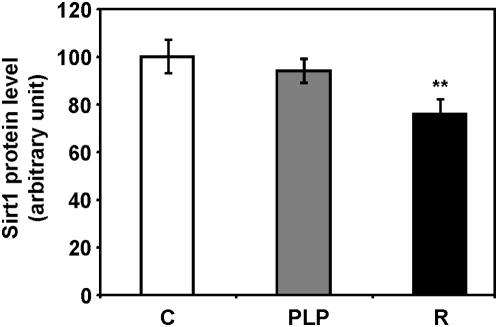
Sirt1 protein levels in muscle. Expression of Sirt1 protein was analysed by Western blotting. Protein levels were expressed as arbitrary units following densitometry analysis (** *P*<0.01 compared to control; n = control (C): 8, postnatal low protein (PLP): 8, recuperated (R): 8).

Sirt1 protein levels in muscle tissue was not affected by postnatal low protein feeding regimen; however, a significant decreased expression was observed in recuperated mice (24% decrease, *P* = 0.007, Figure 5).

## Discussion

Distinct from the widely known and extensively studied CR rodent models in which food is restricted after weaning, lifespan in our rodent models is affected by maternal protein restriction during pregnancy and lactation. Unlike CR rodent models in which lifespan extension is generally observed when CR is initiated regardless of initiation time [2], the maternal protein restriction in our rodent models can increase or decrease lifespan depending on whether the restriction is imposed during pregnancy or during lactation [3], [4]. As maternal protein restricted offspring were weaned onto the same lab chow it is hypothesized that permanent changes in organ structure and/or function occur during this early time period and these changes can assert long term effects on the regulation of lifespan.

Maternal protein restriction applied during suckling to pups born to normally fed mothers significantly retarded their growth. Conversely, pups of low birth weight due to protein restriction during pregnancy underwent rapid catch-up growth when they were suckled by normally fed mothers. We reported previously that growth retardation during lactation was associated with extended lifespan whereas *in utero* growth restriction followed by catch-up growth was associated with shorter lifespan [3], [4]. These associations may be in line with the observation that small body weight in early life is a significant predictor of lifespan with a strong inverse correlation between growth retardation early in life and longevity in a genetically heterogeneous mouse population [31]. Such inverse correlation was found to be strongest for body weights measured early in adult life [31]. In our maternally protein restricted mice we reported previously that PLP mice remained smaller throughout life whereas recuperated mice remained heavier than control animals throughout most of the adult life [32]. Furthermore, genetic mutations in mouse models that lead to increase in lifespan are often associated with dwarfism or reduced body weight [16].

The fact that not all tissue weights were proportionally decreased or increased relative to body weights in PLP and recuperated mice suggests that selective metabolic resource allocation is triggered to maintain the growth of more important tissues, such as brain [7]. Among the tissues examined in PLP mice, brain, lung and thymus showed no reduction in weights as compared to control tissues. When adjusted by body weights brain and thymus in PLP mice were significantly heavier. This may suggest that enhanced functional capacity of brain and thymus is beneficial to health and longevity in mice.

Decreased fasting glucose and insulin concentrations in PLP mice suggest that these animals had a better insulin sensitivity. Improved insulin sensitivity is a frequently observed feature in mouse models with increased lifespan such as those of caloric restriction and genetic mutations. It was observed that calorie restricted rats maintained decreased plasma glucose and insulin concentrations throughout life [33]. Long lived dwarf mice that are deficient in or resistant to growth hormone are hypoinsulinemic and exhibit enhanced whole-animal insulin sensitivity [34], [35], [36]. Moreover, the association between insulin sensitivity and longevity has also been observed in humans as healthy centenarians were found to have a preserved glucose tolerance and insulin action [37], [38]. Our long lived PLP mice therefore bear a common phenotypic characteristic to the long lived dwarf mice and CR rodents and human centenarians. Similarly, we recently observed that PLP rats exhibited significant reduced fasting insulin concentrations at 21 days of age [39].

The better whole body insulin sensitivity in PLP mice was reflected by the protein expression profiles of insulin signalling molecules. Skeletal muscle of PLP mice had significantly higher expression of IRS1 and PKCζ compared to controls. IRS1 belongs to the IRS family of adaptor molecules and is tyrosine phosphorylated in response to the activation of insulin receptor by insulin binding. Tyrosine phosphorylated IRS1 then recruits downstream effector molecules which in turn activate further downstream signalling pathways. IRS1 thus is the initial step and plays a critical role in insulin signalling [40]. The importance of IRS1 in insulin signalling is supported by the fact that decreased levels of IRS proteins, coupled with decreased levels of the IR itself, contribute to the insulin resistance in diabetic states in both rodents and humans [41]. Decreased phosphorylation of IRS1 on Tyr^612^ and Akt on Ser^473^ in the muscle tissue of PLP mice may reflect the fact that the circulating insulin level was significantly lower in these animals. Interestingly phosphorylation of IRS1 on Ser^307^ was also decreased in the muscle tissue of PLP mice as compared to the controls. The phosphorylation on serine residues generally has a negative effect on IRS1 signalling and thus representing a negative feedback mechanism. Indeed serine phosphorylation of IRS1 is generally increased in the insulin-resistant state [40], [42]. PKCζ is another downstream effector in the insulin signalling pathway and plays an important role in activating the glucose transport response [43]. Up-regulation of PKCζ in muscle tissue of PLP mice and IRS1 and reduced serine phoshporylated IRS1 are thus consistent with the enhanced insulin sensitivity at the whole animal level.

In contrast to PLP mice, protein expression levels of insulin signaling molecules in the muscle tissue of recuperated mice were generally decreased. Significant decreases in protein levels of PKCζ and PI3 kinase subunits p85 and p110β suggests a reduced insulin signaling capacity. Indeed phosphorylation of IRS1 on Tyr^612^ was significantly reduced compared to the controls although fasting insulin levels in recuperated mice were similar to that of controls. In addition, Ser^473^ phosphorylated Akt was also significantly reduced compared to the controls. These observations indicate that low birth weight followed by rapid catch-up growth is associated with reduced levels of key insulin signaling proteins. It is well documented that individuals with low birth weight coupled with rapid catch-up growth are at increased risk of developing insulin resistance and type 2 diabetes [44]. We previously demonstrated that maternal protein restriction in rats leads to fetal growth restriction, insulin resistance and type 2 diabetes [45]. This is associated with specific changes in expression of components of the insulin-signaling pathway including reduced expression of PKCζ and the p110β catalytic subunit of PI3 kinase [46]. We also showed that young men who had a low birth weight have strikingly similar alterations in insulin signaling molecules in muscle and fat [47], [48]. These findings provide strong evidence for the importance of maternal diet in mediating the relationship between poor early growth and subsequent risk of diabetes. Our current findings in mice and recent observations in rats [39] suggest that reduced expression of these key signaling molecules can be detected at an early age and could provide a molecular fingerprint for later adult diseases such as type 2 diabetes.

Sirt1 is one of sirtuin family proteins which are conserved NAD^+^-dependent histone deacetylases with broad biological functions [49], [50]. Sirt1 affects many metabolic and stress resistance pathways including those involved in DNA repair, apoptosis, glucose and fat metabolism [51]. In particular, Sirt1 plays a pivotal role in mediating effects of CR on lifespan extension [52]. The levels of Sirt1 have been reported to increase in rodent and human tissues in response to CR and this increase can trigger favourable changes in metabolism and enhanced stress tolerance [53], [54]. This was supported by findings that transgenic mice overexpressing Sirt1 demonstrated a phenotype resembling caloric restriction [29] although whether these mice have an extended lifespan remains to be established. Sirt1-null mice were also shown to have lost the normal metabolic response to CR and failed to show lifespan extension by CR [30]. It is thus surprising that Sirt1 level showed no increase in muscle tissue of PLP mice that live longer. This may be due to tissue specific regulation of Sirt1 expression in response to maternal protein restriction as an up-regulation of Sirt1 expression can be detected in the kidneys of PLP mice (unpublished data). Indeed tissue-specific regulation of Sirt1 was observed in CR mice in which both the protein level and activity of Sirt1 in the liver were down-regulated compared to the *ad libitum* fed controls [55]. However, the decrease in Sirt1 protein in muscle tissue of recuperated animals which have a 26% decrease in mean lifespan [4] is in line with its role in regulation of lifespan and may thus have a negative impact on longevity in these animals.

In summary, we have shown that maternal protein restriction during early life can influence growth rate of mouse offspring in the first three weeks of their life. The change in the whole body growth is not proportionately reflected by individual organ weights suggesting selective organ growth occurred to spare growth of vital organs such as the brain. At the molecular level, alteration in protein expression of insulin signalling molecules was detected at 21 days of age. Up-regulation of IRS1 and PKCζ in the muscle tissue of PLP mice might underlie the improved insulin sensitivity which in turn might contribute to the lifespan extension in these animals. Conversely, low birth weight followed by rapid catch up growth was associated with down-regulation of insulin signalling proteins which may predispose these animals to insulin resistance later in life. Decreased expression of Sirt1 in the muscle of recuperated animals further provided a molecular fingerprint that is indicative of a shortened lifespan. Taken together, this study suggests that in response to maternal protein restriction remodelling in organ growth and molecular expression occur at an early age that will have lasting long term effects and ultimately influences lifespan.

## Materials and Methods

### Animals

Mice (C57/b16) were bred locally at a designated animal unit of the University of Cambridge (Cambridge, UK). Adult female mice were housed individually and were maintained at 22°C on a 12∶12 hours light∶dark cycle. They were mated when they were 6 weeks old (∼22 g in body weight) and assumed to be pregnant when a vaginal plug was expelled. Dams were fed *ad libitum* either a control diet (containing 20% protein) or an isocaloric low protein diet (containing 8% protein) during gestation and lactation. Both the control and low protein diet were purchased from Arie Blok (Woerden, the Netherlands) and detailed diet composition is provided in Table 2. Cross-fostering techniques were used at 3 days of age to establish three groups: control (offspring born to and suckled by control diet fed dams); postnatal low protein (PLP) (offspring of control dams nursed by low protein diet fed dams); and recuperated (offspring of low protein diet fed dams nursed by control dams). A low protein diet during pregnancy had no statistically significant effect on litter size (*P* = 0.11): pups per litter: low protein, 6.7±0.4; control, 7.4±0.4. As described previously [4], to maximize the effects of maternal diet differences on offspring growth, recuperated pups were culled to 4 (to maximize growth during suckling) and control pups were culled to 8 (if the number of pups was greater than 8) whereas PLP pups were unculled. Litter size standardization was carried out randomly. Body weights of animals were recorded at days 3, 7, 14 and 21 of age. At day 21 pups were removed from dams and starved overnight before glucose and insulin measurements and organ/tissue collections. One male was selected at random from each litter in the current study. Fasting blood was collected following decapitation, allowed to clot for 30 min, and then centrifuged at 7,200 g for 3 min to obtain serum. Organs/tissues were weighed, snap frozen in liquid nitrogen and stored at −80°C until use. All animal procedures were carried out in compliance with the British Home Office Animals Act (1986).

**Table 2:** Detailed nutrient components of the control (20) and low protein (8%) diets.

Dietary component	20%	8%
Minerals and vitamins	5.35	5.45
Casein, %(88 g protein/100 g)	22.00	9.00
DL-Methionine, %	0.20	0.08
Corn starch, %	8.00	8.00
Cellulose	5.00	5.00
Soybean oil, %	4.30	4.30
Cerelose/dextrose, %	55.15	68.17

### Glucose and insulin measurements

Fasting blood glucose was measured using a blood glucose analyzer (Hemocue, Angelholm, Sweden). Serum insulin concentrations were measured using a mouse insulin ELISA kit (Mercodia Ultra-sensitive Mouse Insulin ELISA, Mercodia, Uppsala, Sweden). All samples were assayed in duplicate, and an intra-assay coefficient of variation of up to 5% was accepted.

### Western blotting

Total protein was extracted from mouse muscle (vastus lateralis) by homogenization in ice-cold lysis buffer [50 mM HEPES (pH 8), 150 mM NaCl, 1% Triton ×100, 1 mM sodium orthovanadate, 30 mM NaF, 10 mM sodium pyrophosphate, 10 mM EDTA, and a protease inhibitor cocktail set III (Calbiochem Novabiochem Biosciences, Nottingham, UK)]. The total protein concentration in the lysates was determined using a Sigma copper sulfate/bicinchoninic acid assay. Samples were diluted to a concentration of 2 mg/ml in Laemmli's buffer. Twenty five micrograms of total protein were subjected to SDS-PAGE and transferred onto PVDF immobilon-P membrane (Millipore, Billerica, MA, USA). Membranes were blocked in Tris-buffered saline with 0.1% Tween-20 and 5% dry milk, and incubated with the primary antibodies against: insulin receptor-β (IR-β), IGF1 receptor (IGF1R), protein kinase C zeta (PKCζ and PI3-kinase p110β from Santa Cruz Biotechnology (Santa Cruz, CA, USA), Akt1, Akt2 and phospho-Akt (Ser^473^) from Cell Signaling (Beverly, MA, USA); PI3-kinase p85α, IRS1, phosphor-IRS1 (Tyr^612^), phosphor-IRS1 (Ser^307^) and Sir2 from Upstate Biotechnology (Millipore, Billerica, MA, USA). The bound primary antibodies were detected by horseradish peroxidase-conjugated secondary antibodies (Amersham Biosciences, Piscataway, NJ, USA and Dako, Glostrup, Denmark), followed by enhanced chemiluminesence (Amersham Biosciences). The densities of the bands were quantified using an Alpha Imager (Alpha Innotech Corporation, San Leandro, CA).

### Statistical analysis

Nonparametric data were log transformed prior to testing and are shown as the geometric mean (95% confidence intervals). Parametric data are expressed as mean±SEM. Data were analyzed using a one-way ANOVA with maternal diet as the independent variable factor. When the effect of maternal diet was significant, Duncan's post hoc testing was used to analyze significance differences between groups. A *P*<0.05 was considered statistically significant.
